# Testing functional anchor groups for the efficient immobilization of molecular catalysts on silver surfaces

**DOI:** 10.1038/s42004-024-01186-3

**Published:** 2024-05-10

**Authors:** Ole Bunjes, Alexandra Rittmeier, Daniel Hedman, Shao-An Hua, Lucas A. Paul, Franc Meyer, Feng Ding, Martin Wenderoth

**Affiliations:** 1 IV. Physikalisches Institut, Georg-August-Universität Göttingen, Friedrich-Hund-Platz 1, 37077 Göttingen, Germany; 2 Center for Multidimensional Carbon Materials, Institute for Basic Science (IBS), Ulsan, 44919 Republic of Korea; 3 Institut für Anorganische Chemie, Georg-August-Universität Göttingen, Tammannstraße 4, 37077 Göttingen, Germany; 4 International Center for Advanced Studies of Energy Conversion (ICASEC), Georg-August-Universität Göttingen, D-37077 Göttingen, Germany; 5 Department of Materials Science and Engineering, Ulsan National Institute of Science and Technology (UNIST), Ulsan, 44919 Republic of Korea

**Keywords:** Surface assembly, Scanning probe microscopy, Surfaces, interfaces and thin films, Molecular self-assembly

## Abstract

Modifications of complexes by attachment of anchor groups are widely used to control molecule-surface interactions. This is of importance for the fabrication of (catalytically active) hybrid systems, viz. of surface immobilized molecular catalysts. In this study, the complex *fac*-Re(^S-S^bpy)(CO)_3_Cl (^S-S^bpy = 3,3′-disulfide-2,2′-bipyridine), a sulfurated derivative of the prominent Re(bpy)(CO)_3_Cl class of CO_2_ reduction catalysts, was deposited onto the clean Ag(001) surface at room temperature. The complex is thermostable upon sublimation as supported by infrared absorption and nuclear magnetic resonance spectroscopy. Its anchoring process has been analyzed using scanning tunneling microscopy (STM) and density functional theory (DFT) calculations. The growth behavior was directly contrasted to the one of the parent complex *fac*-Re(bpy)(CO)_3_Cl (bpy = 2,2′-bipyridine). The sulfurated complex nucleates as single molecule at different surface sites and at molecule clusters. In contrast, for the parent complex nucleation only occurs in clusters of several molecules at specifically oriented surface steps. While this shows that surface immobilization of the sulfurated complex is more efficient as compared to the parent, symmetry analysis of the STM topographic data supported by DFT calculations indicates that more than 90% of the complexes adsorb in a geometric configuration very similar to the one of the parent complex.

## Abstract (web summary)

Thiolate groups are promising anchors to immobilize catalysts, particularly on coinage metal surfaces, but the anchor group’s impact on the local adsorption configuration of the catalyst is often overlooked. Here, the authors study a sulfurated derivative of the prominent Re(bpy)(CO)3Cl carbon dioxide reduction catalysts on a Ag(001) surface at room temperature.

## Introduction

Surface anchoring groups for molecular catalysts are deliberately chosen, usually based on electronic considerations for controlling molecule-surface interactions. Conceptually, anchors are employed for immobilization and coupling of adatoms^1^ or molecules on and to surfaces. More technically, this is used, e.g., for hydrogen storage^2^, dye-sensitized solar cells^3^, or for single-molecule electronics^4–7^. For the design of highly functional catalytically active hybrid systems, the employment of rational anchor groups is seen as a promising strategy^8–12^. Manipulation of the anchor geometry^13^, changing the number of contact points^14,15^, the linker length^16^, as well as fully exchanging the anchor group can strongly affect the molecule-surface interaction^17^ and electron transfer rates^18^.

**Fig. 1:**
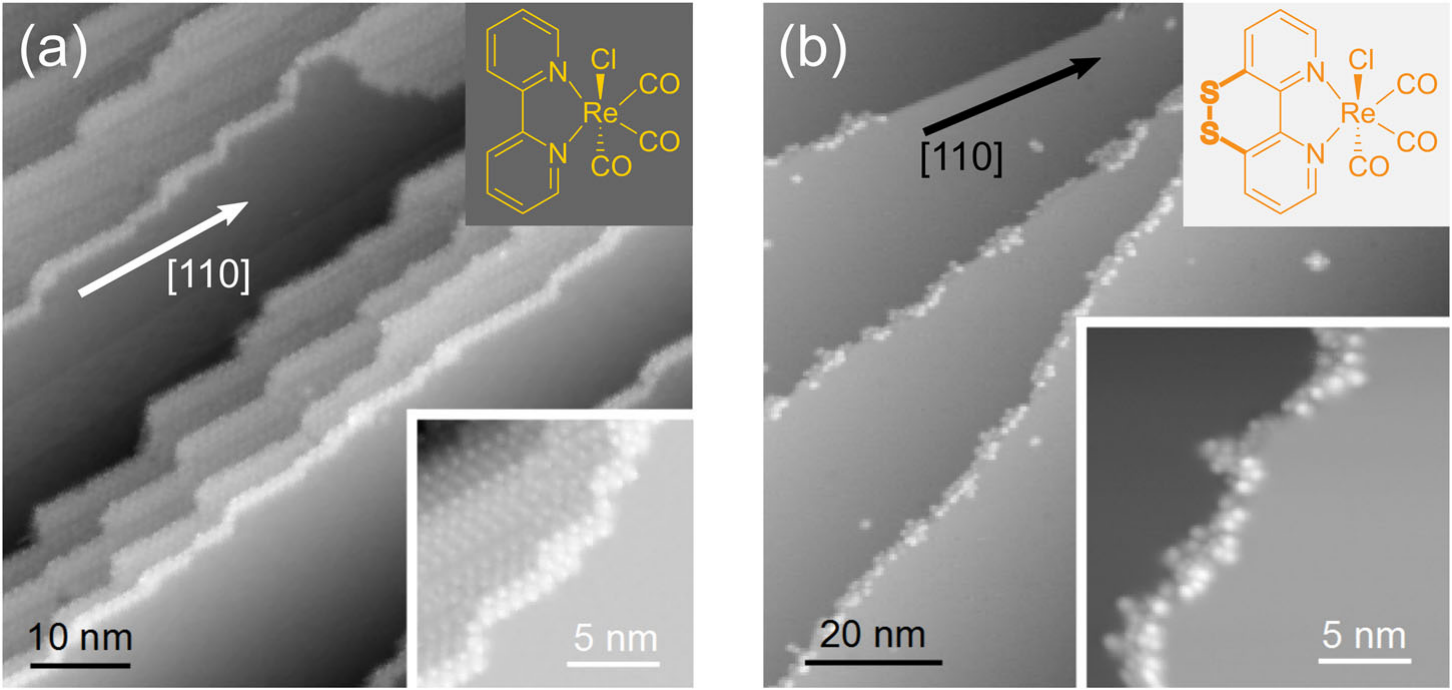
Contrasting the surface adsorption of Rebpy^S-S^ and of Rebpy on the Ag(001) surface. **a** Step edges with a preferential orientation along the silver crystal’s [110] orientation and terraces covered by long-range ordered Rebpy structures. *U*_bias_ = 2 V, *I*_set_ = 50 pA. **b** Randomly oriented step edges covered by disordered Rebpy^S-S^-structures. *U*_bias_ = 0.2 V, *I*_set_ = 50 pA. The corresponding structural formulas of the molecules are given as upper insets. The lower insets show high-resolution topographies of step-edge-decorating molecular structures. Data are colored such that the step edges are well visible.

Very recently, a functionalized derivative of the archetypical rhenium complex *fac*-Re(bpy)(CO)_3_Cl (short Rebpy) that is a well-known CO_2_ reduction catalyst^19^, viz. the sulfurated *fac*-Re(^S-S^bpy)(CO)_3_Cl (^S-S^bpy = 3,3′-disulfide-2,2′-bipyridine^20^) (short Rebpy^S-S^) was introduced^21,22^. It features a disulfide bond on the backside of the bipyridine ligand, as depicted in the upper inset of Fig. 1b. We have established the thermochemistry of the disulfide/dithiol interconversion and its ligand-based proton-coupled electron transfer chemistry, as well as the homogenous electrochemical CO_2_ reduction^21^, while Cattaneo et al.^22^ have demonstrated the capability of attaching the Rebpy^S−S^ to a gold film electrode surface via the peripheral sulfur anchoring groups on the ligand moiety. Based on a sum frequency vibrational spectroscopy (SFG) study, averaging over large surface areas, in combination with density functional theory (DFT) calculations, an energetically most favorable configuration in which dithiolate-gold bonds form upon complex adsorption has been proposed that appears sterically favorable for heterogeneous CO_2_ reduction catalysis. The utilization of thiolate groups as anchors seem promising, particularly for coinage metal surfaces, but their impact on the local scale adsorption configuration of Rebpy^S−S^ on a surface, i.e., taking into account surface defects for example, step edges, remains unclear.

In the present work, we have investigated the effect of a disulfide bond at the backbone of a bipyridine ligand on molecular surface adsorption using Rebpy^S-S^ as a model complex on the clean Ag(001) surface at room temperature. The molecular structure is analyzed by means of scanning tunneling microscopy (STM) operating at low temperatures of 77 K under ultrahigh vacuum (UHV) conditions as well as by DFT calculations. Atomic scale analysis enables us to evaluate the anchoring process of the molecules. We, therefore, provide evidence that as a consequence of the anchor group, the molecule-surface interaction is strengthened and the majority of the Rebpy^S-S^ complexes, are found to anchor in a configuration similar to the one reported for the parent complex Rebpy^23^ that was recently studied on identical surfaces.

## Results and discussion

The goal of this work is to evaluate the effect of the sulfur anchors at the ligand periphery of *fac*-Re(^S-S^bpy)(CO)_3_Cl (^S-S^bpy = 3,3′-disulfide-2,2′-bipyridine), short Rebpy^S-S^, on the molecule-surface interaction as compared to the parent complex *fac*-Re(bpy)(CO)_3_Cl (bpy = 2,2′-bipyridine), short Rebpy, on the Ag(001) surface, which we have studied earlier^23^. For the sulfurated complex, we used very similar sample preparation and investigation methods for direct comparison (see ‘Methods’), which allows us to attribute observed differences in the growth behavior to the sulfur anchors. After proving its chemical stability upon sublimation, the complex was sublimed onto freshly cleaned Ag(001) surfaces at room temperature and studied at low temperatures by means of STM. The local adsorption geometry was investigated with the help of DFT calculations. Figure 1 shows two exemplary topographies of molecule-covered silver terraces, one for each of the deposited molecular species. For the adsorption of the parent complex (Fig. 1a), the following aspects are characteristic^23^. Single molecules were neither found on the free terraces nor attached to step edges. Instead, nucleation was exclusively found to occur at oriented step-edge segments aligned along the crystal’s high symmetry direction [110]. Further occupation of the step edges was found to be accompanied by reorienting of misaligned step edges. Consequently, the overwhelming majority of the decorated step edges visible in Fig. 1a are oriented along [110]. Nucleation of molecular monolayers is found to take place at well-oriented and covered step edges, and the layers show intrinsic long-range order of certain complexity^24^. Figure 1b shows a silver surface decorated by the sulfurated complex Rebpy^S-S^. Starting with the investigation of the chemical stability of the complex undergoing the sample preparation procedure, its growth behavior will be studied in detail in the following.

### Stability of the complex upon sublimation

**Fig. 2:**
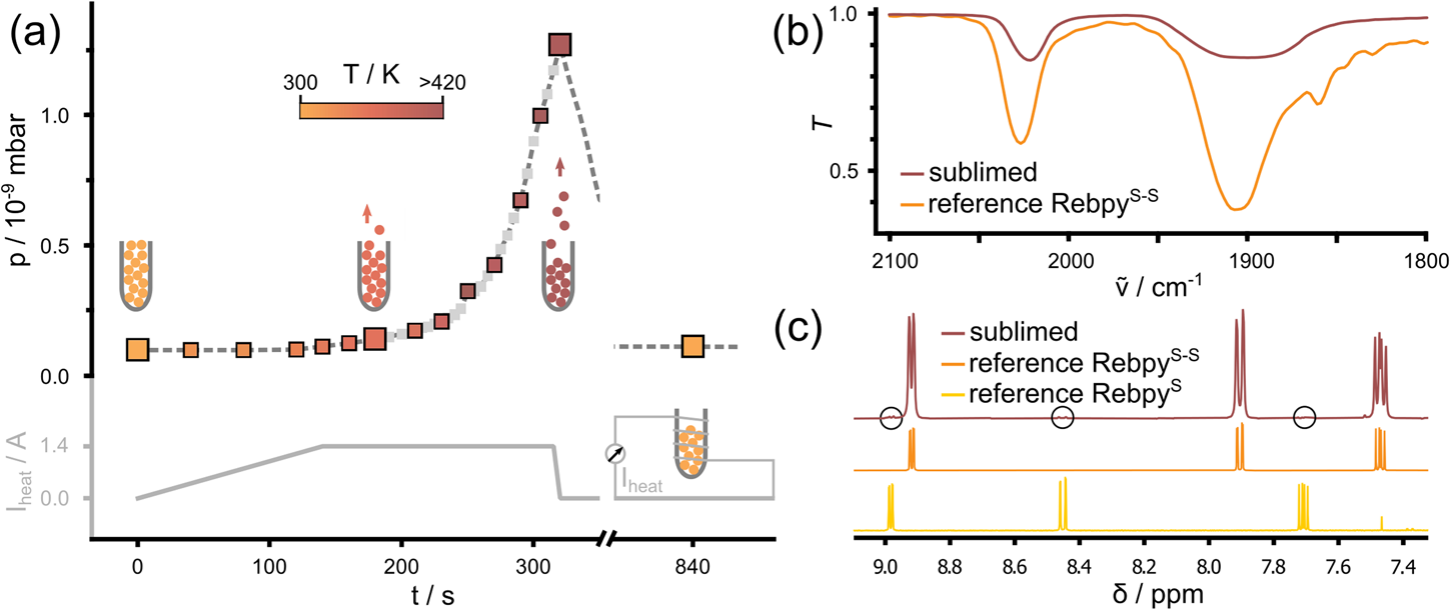
Characterization of RebpyS-S for sample preparation. **a** Sublimation protocol. The heating current is regulated as a function of time, causing a color change of the molecular powder as well as giving rise to a pressure increase in the chamber. The insets depict the source, and the colors are chosen in correspondence to the color of the molecular material shown in Supplementary Fig. 1. **b** IR measurements of the complex directly after synthesis (orange) and after sublimation onto glass at 200 °C (dark red). **c** ^1^H NMR spectra (solvent CDCl_3_) of the molecular powder taken from the molecule source after sample preparation (dark red) with corresponding reference spectra. A high-resolution version of dark red spectrum with quantitative evaluation of peaks encircled in black is shown in Supplementary Fig. 2.

The complex Rebpy^S-S^ was sublimed onto the clean Ag(001) surface, following the sublimation protocol shown in Fig. 2a and using the molecular source sketched as inset. Details on the preparation can be found under ‘Methods’. Atomic scale data of the prepared surfaces were acquired in a home-built STM at 77 K. As can be seen in the photos of the compound in Supplementary Fig. 1, the color of the molecular material changes upon heating. Thermochromism is a common phenomenon for molecular transition metal complexes in solid state^25^. In case of Rebpy^S-S^, reversibility of the color change indicates that it is not associated with degradation of the molecular species. To further rule out the color change to be caused by an irreversible chemical change, we evaluated the stability of the complex during the sample preparation procedure using two complementary spectroscopic characterization techniques. Infrared (IR) absorption spectroscopy is used to monitor the characteristic CO absorption bands that are, due to the local position of the CO groups within the molecule, very sensitive to the co-ligands at the rhenium center. The IR signatures of freshly synthesized as well as of material sublimed at about 200 °C under pressures *p* < 10^−3^ mbar onto glass are shown in Fig. 2b. Both spectra show the characteristic CO absorption fingerprint of the *fac*-{Re(CO)_3_} moiety in Rebpy^S-S^, confirming that the molecular complex remains intact upon sublimation. In addition, proton nuclear magnetic resonance (^1^H NMR) spectroscopy was applied to identify the proton resonances of the sulfurated bipyridine ligand of Rebpy^S-S^. We have analyzed the molecular material that has remained inside the source after several sample preparations, i.e., after having been heated to temperatures >150 °C nine times under pressures *p* < 5 × 10^−8^ mbar and having been stored under ambient conditions for a few months. The ^1^H NMR spectrum is shown in Fig. 2c. The dominant peaks agree with the spectrum of authentic Rebpy^S-S^ given as reference. Rather small peaks (encircled in black) belong to the closely related complex *fac*-[Re(^S^bpy)(CO)_3_Cl] (with ^S^bpy = thieno[3,2-*b*:4,5-*b*′]dipyridine) that has lost one sulfur atom, short Rebpy^S^. A high-resolution image of the spectrum, as well as the integration of the ^1^H NMR spectrum, can be found in Supplementary Fig. 2, indicating a minor proportion of only 2–3% of Rebpy^S^, likely the result of thermal degradation^21^. This corroborates the complexes to be fairly stable upon thermal treatment and sublimation.

### Immobilization of Rebpy^S-S^ on Ag(001)

In the following the surface adsorption of Rebpy^S-S^ is investigated.

**Fig. 3:**
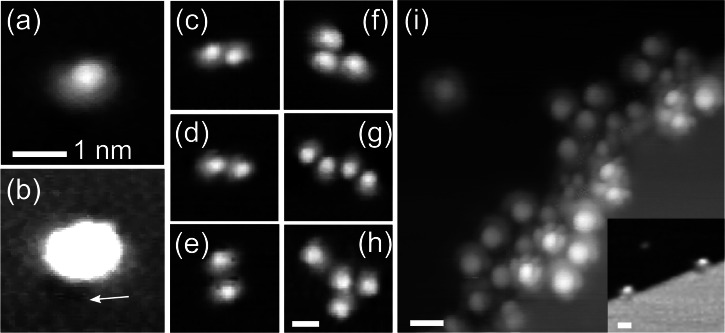
Immobilized molecules. **a** Topography of a single molecule on the free surface. **b** Same topography as **a**, but with shifted color scale. The white arrow indicates a surface defect. **c**–**h** Differently sized molecular clusters on the free terrace. **i** Molecules clustering at a step edge. Inset: single molecules attached to a step edge. All scale bars have a length of one nanometer. Tunneling parameters: **a**–**e** *U*_bias_ = 1 V, *I*_set_ = 50 pA. **f** *U*_bias_ = −1 V, *I*_set_ = 50 pA, **h**–**i** *U*_bias_ = 0.4 V, *I*_set_ = 50 pA, inset **i** *U*_bias_ = 1 V, *I*_set_ = 50 pA.

As evident by Fig. 1b, molecules are dominantly (~90%) found to be attached to step edges or to molecular clusters decorating step edges (cf. Fig. 3i). Also, a small minority of single molecules are found on the free terraces, Fig. 3a, where careful analysis reveals that this always goes along with point defects in/on the surface in direct proximity Fig. 3b displays a corresponding tiny topographic depression close to the molecule that is indicated by the arrow. We found some evidence suggesting these could be sulfur impurities on/within the surface^26^ originating from the surface preparation procedure^27^, details in Supplementary Figs. 3–5. The summary of our statistical analysis can be found in Supplementary Note 1. Notably, these defects are not found next to molecular clusters of two or more molecules, which is another option to find molecules on the free terraces, Fig. 3c–h. We conclude that single molecules are rather mobile on the undisturbed silver terrace, requiring a nucleus to consist of more than a single molecule, with either the attachment to a surface defect or the attachment to other molecules leading to immobilization.

### Structure of molecular clusters

The investigation of the molecular arrangement within the molecular clusters requires a detailed analysis of the appearance of single molecules. The topographic contour found for the vast majority of complexes shows asymmetry, as the one shown in Fig. 3a. Supplementary Fig. 6 shows the proposed orientation of the molecule within some clusters in more detail. We would like to mention that a small fraction of the molecules in the single-digit percentage range does exhibit different topographic appearances (e.g., top left-hand corner of Fig. 3i), which may be issue to a separate study. The majority is characterized by an asymmetric appearance with a topographic maximum (‘head’) on one side and a plateau region (‘tail’) on the opposite side. This unit is found to be the building block underlying the molecular clusters shown in Fig. 3c–i. Closer inspection of molecular clusters reveals that different alignments of these units, i.e., neighboring molecules, are possible (see Fig. 3c–e). For example, we find the configurations shown in d and e in which the two molecules are attached to each other in a head-to-tail (maximum-to-plateau) and a tail-to-tail fashion, respectively. The configuration head-to-head has not been observed, indicating this to be energetically less favorable. In Fig. 3f–h, molecular clusters consisting of several molecules are shown. From our data, we conclude that the geometric relations found for clusters consisting of two molecules can be regarded as the basic building blocks and transferred to larger clusters. For example, the four-molecule cluster given in Fig. 3g is build up from two identical copies of the two-molecule cluster shown in Fig. 3c, while the cluster shown in Fig. 3h is a combination of the two-molecule relations found in 3d, e. In Fig. 3i molecules clustering at a step edge can be seen. Importantly, the orientations of the molecular contours (head-and-tail) are found to rotate with respect to the surface normal within the same image indicating the visual impression not to originate from a tip artifact. Again, the same observations can be made, e.g., two molecules in the upper right-hand corner of the image show a relation similar to the one seen in Fig. 3c and several other molecules align in a head-to-tail fashion with respect to their neighboring molecules, as do the two molecules in the cluster shown in Fig. 3d. These observations indicate that inside clusters there are preferential alignments of neighboring molecules with respect to each other.

### Anchoring model for Rebpy^S-S^ on Ag(001)

**Fig. 4:**
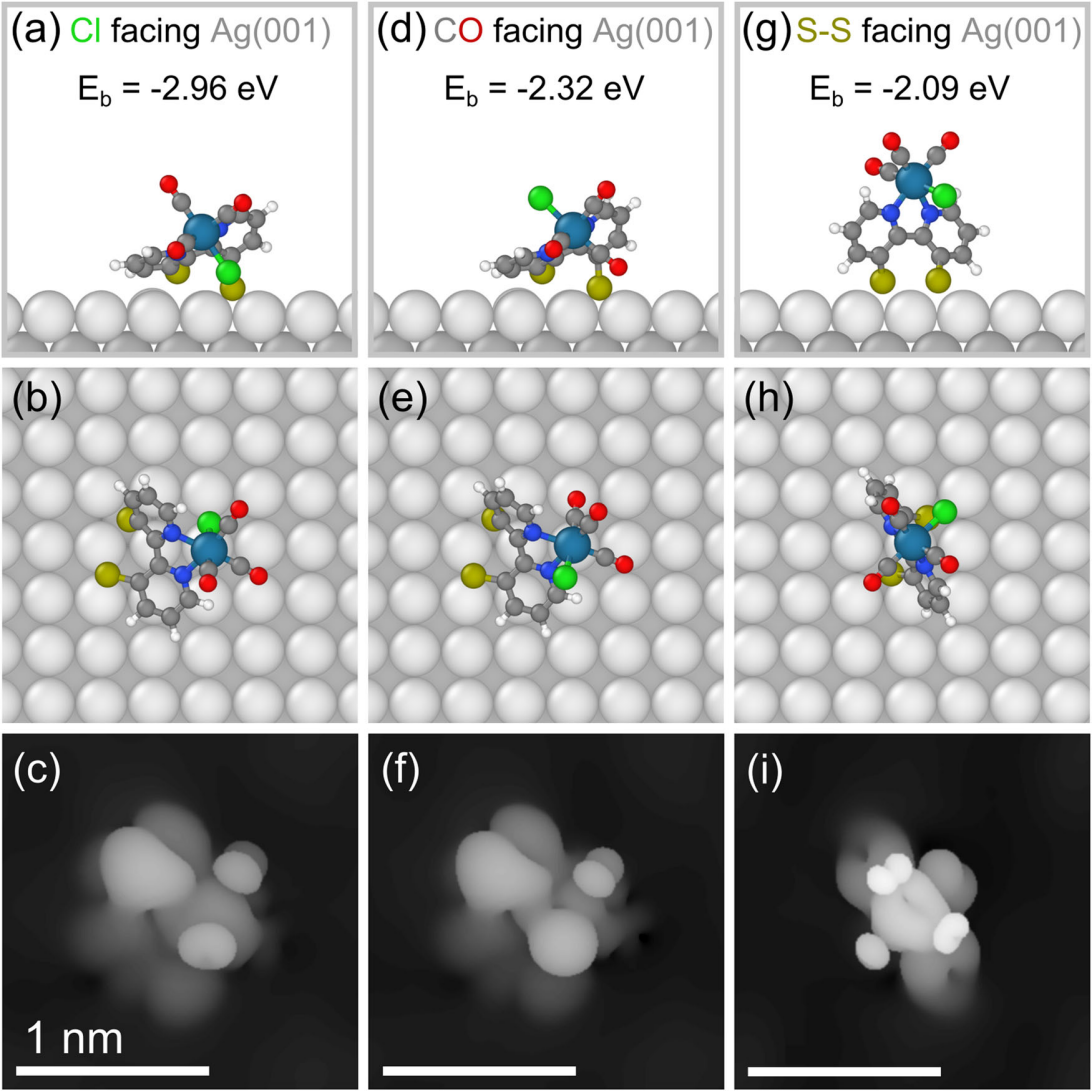
DFT calculated adsorption configurations. **a** Adsorption configuration of molecule with the Cl ligand facing the substrate in side view, **b** in top view. The binding energy *E*_b_ is given above. Color code: white = H, dark gray = C, blue = N, red = O, yellow = S, green = Cl, light gray lattice = top layer Ag, dark gray lattice = second layer Ag, turquoise = Re. **c** Simulated constant current topography, details in ‘Methods’. **d** Adsorption configuration of molecule with the CO group facing the substrate in side view, **e** in top view. **f** Simulated constant current topography. **g** Adsorption configuration of molecule with the sulfur anchors facing the substrate in side view, **h** in top view. **i** Simulated constant current topography. Tunneling parameters of the simulated topographies: *U*_bias_ = 1 V, *I*_set_ = 50 pA. All scale bars have the length of one nanometer. Images were generate using the OVITO software package^48^.

In order to investigate the adsorption geometry of the single molecules we complemented our experimental results by theoretical DFT calculations. We studied three different adsorption configurations that can be seen in Fig. 4. As for the parent complex we considered the two configurations shown in Fig. 4a, d in which the molecule adsorbs ‘flatly’ on the surface, once facing the substrate with its chloride ligand and once with the opposing CO group. And, inspired by the work of Cattaneo and co-workers^22^, we also studied the configuration they found to be energetically most favorable on a gold surface, in which the molecule adsorbs in a standing upright configuration with the sulfur anchors facing the substrate, Fig. 4g. Although before relaxation all molecules have intact disulfide bonds, after relaxation the three different configurations have in common that the disulfide bond is broken. This indicates that the interactions between sulfur and silver atoms are energetically more favorable in the case of the broken disulfide bond as compared to the interaction between the intact disulfide group and the silver substrate. This is in accordance with the known affinity of thiolates for silver (and gold) surfaces, and with the experimental observation that the S-S bond of organic disulfides evaporated onto silver is cleaved on adsorption to form thiolate films on the silver surface^28,29^. The breaking of the S-S bond in Rebpy^S-S^ is likely to result from a combination of both the charge transfer between molecule and substrate as well as the covalent interaction between the sulfur and the silver atoms (Supplementary Fig. 7). For completeness, the calculated electronic states of the molecule are shown in Supplementary Fig. 8. In descending order the standing configuration is least energetically favorable of the three configurations. The configuration with the CO groups facing the surface shows a lower binding energy, but the most stable configuration is the one in which the chloride ligand faces the underlying silver surface. Therefore, the sulfur-silver interaction seems to contribute similarly to the surface binding for all three configurations. In case of the two lying configurations additional contributions result from the interactions of the silver surface with the CO and the Cl ligands, with the latter being energetically more favorable, which goes well along with chemical intuition. The most stable configuration is similar to the configuration found to be most stable for the parent complex (except for the sulfur anchors). To correlate the experimental results with the calculated adsorption configurations, we simulated constant current topographies using the Tersoff-Hamann scheme^30^. These are shown in Fig. 4c, f, i. While the topography of the standing configuration shows two quasi-symmetry axes the two other topographies are way less symmetric with one rather dominant topographic maximum on one of its sides that from its spatial position coincides with the tilted bipyridine unit, for better comparison Figs. 3a, 4c, f are given side-by-side in Supplementary Fig. 9. This indicates that the standing configuration can be excluded as most probable cause of the asymmetric appearance observed in the STM topographies. Furthermore, the binding energy indicates the lying configuration with the Cl facing the substrate to be the most reasonable candidate for the observed asymmetric topographic contour. With this, the avoidance of the head-to-head configuration inside the molecular clusters translates into an avoidance of two sulfurated backbones being close to each other. This could result from repulsive dipole-dipole interactions of the surface-bound bipyridine dithiolato ligands of two neighboring molecules due to charge transfer between the molecule and the surface upon adsorption, Supplementary Fig. 7.

### Comparing the growth of Rebpy and of Rebpy^S-S^ on Ag(001)

In order to evaluate the effect of the anchor groups at the backbone of the bipyridine ligand, the growth observed for the parent complex in ref. ^23^ is directly contrasted to the growth observed for the sulfurated derivative.

Starting with the single-molecule DFT calculations, the parent complex Rebpy has its energetically most favorable alignment with the chloride ligand facing the substrate. On the free terrace, this configuration has a binding energy of −1.86 eV. A single complex with the chloride ligand attached to a [110] step edge has a lower binding energy of −3.24 eV^23^, which therefore is significantly more favorable. In comparison, all three different adsorption geometries studied for the sulfurated complex Rebpy^S-S^ on the free terrace have binding energies smaller than −2.09 eV. These are lower (indicating stronger binding) than for the individual parent complex on the free terrace but are higher (weaker binding) than for the parent attached to a step edge. Nonetheless, on the free terrace for both complexes it is found to be energetically most favorable to adsorb in a lying configuration with the chloride ligand facing the substrate. The binding of the sulfurated complex Rebpy^S-S^ is about one eV stronger than for the parent Rebpy, indicating a significant contribution from the sulfur anchors, making the molecule’s surface adsorption energetically more favorable.

Experimentally, the parent complexes are found to nucleate in small clusters of molecules at specifically oriented surface step edges ([110] direction of the substrate crystal)^23^. Interestingly, for the sulfurated complex Rebpy^S-S^ substrate steps along the direction [110] appear to be rather unlikely to be occupied as can be seen in Figs. 1 and 3 (topmost step edge in Fig. 1b, inset of Fig. 3i). Nucleation of the parent complex was found to occur in clusters of molecules at oriented step edges and at covered and oriented step edges. In contrast, the sulfurated complexes nucleate at several different surface sites: at surface point defects, at other molecules on the free terraces or at surface step edges, or at molecules attached to step edges. While for the immobilization of the parent complex both the interaction with the surface steps and the neighboring molecules is necessary, the sulfurated complexes are immobilized already once attached to a surface defect or a second molecule, both of which indicate a stronger molecule-surface (as supported by the DFT results), molecule-defect and molecule-molecule interaction for the latter. These differences must originate in the different nature of the interactions between the molecule and the surface and in between the molecules. For nucleation of the parent Rebpy, several molecules must form a cluster at the well-oriented step-edge segments along [110]. Hence, single molecules diffusing to these sites without finding other molecules are not stable and, therefore, are not immobilized. If several molecules attach there simultaneously and interact with each other, these oriented step edges provide a suitable environment for the alignment of the molecules such that their interaction immobilizes the small cluster. It is likely that also the sulfurated complex Rebpy^S-S^ is not stable as a single molecule at these oriented step edges but instead is rather mobile. In contrast to the parent Rebpy, the sulfurated complex sticks to disordered step-edge regions already as single molecule. This could reduce the likelihood of attachment of at least two molecules at an ordered segment at the same time, thereby preventing their interaction and possible immobilization resulting in non-decorated step-edge segments along [110].

For the parent complex, step-edge decoration is accompanied by restructuring of the step edges along the very same crystal direction, while no indication of something similar is found for the sulfurated complex. The delicate interaction between the parent complex and the oriented step edges could be the reason for the restructuring of the steps that is observed for decorated step edges. It is energetically favorable for the molecules to take a certain geometric relation with respect to the neighboring molecules. The likelihood for structural correspondence seems to be increased at oriented step edges, which is why nucleation happens there. If now by chance, e.g., through silver atom diffusion, directly neighboring step-edge segments align along this crystal direction, this would increase the likelihood for attachment of further molecules. As a result, both the cluster as well as the length of the oriented segment would increase. For the sulfurated complex Rebpy^S-S^, the interaction between the molecule and surface step is stronger, which is why it already attaches as a single molecule at randomly oriented step edges. This stronger interaction might hinder silver atom diffusion at the covered step edges, which would explain that no reorienting occurs.

Going to larger molecule clusters, the parent complex is found to show exceptionally highly ordered cluster growth, with the neighboring molecules adopting rather well-defined orientations with respect to each other, both in molecular chains and inside monolayers^23^. While the sulfurated complex shows some preferential neighboring alignments inside molecule clusters as well, its growth mode is completely different, and no comparable order has been observed inside larger clusters that are attached to step edges, cf. Fig. 3i. This indicates that the immobilizing molecule-molecule interaction is less sensitive to the molecule orientation for the sulfurated complex. We make the reliance of the parent complex on specific interactions with several directly neighboring molecules for immobilization responsible for its ordered growth. Non-aligned molecules are not immobilized by the clusters, which results in ordered clusters attached to step edges, the nucleation of the monolayer at ordered and decorated step-edge segments, and highly ordered monolayer growth. For the sulfurated derivative, on the other hand, the interaction between two neighboring molecules is sufficient for immobilization on the free surface. Furthermore, the functional anchor group reduces the molecules’ dependence on perfect alignment with respect to a neighboring molecule. This results in many different geometries that allow for immobilizing interaction, as evidenced by the different geometric relations between neighboring molecules found in many-molecule clusters on the free terraces and in clusters of molecules attached to step edges, cf. Fig. 3. In this way, the growth of the sulfurated complex works without the requirement of high intrinsic order.

## Conclusion

In this study, we have investigated the immobilization process of Rebpy^S-S^ equipped with sulfur anchors on the bpy ligand periphery to the Ag(001) surface at room temperature on the atomic scale. Direct comparison between the adsorption of two closely related molecules that only differ by the attachment of a functional sulfur-based anchor group at the molecule’s periphery allows to attribute observed differences in the anchoring process unambiguously to the attachment of the anchor group. It is found that the sulfur anchors drastically alter the molecule-surface interaction comparing the growth of the parent complex Rebpy to the one of its sulfurated derivative Rebpy^S-S^. A strengthening of the molecule-surface interaction becomes evident as the derivative shows nucleation of single molecules at surface defects and at step edges, which is not observed for the parent system. And, while immobilization of the parent complex relies on very defined geometrical alignment with its neighboring molecules, this prerequisite is found unnecessary for the derivative. In this way, the sulfur substituents are found to indeed serve as anchoring groups. This is further supported by the stronger binding between the sulfurated complex and the silver surface found using DFT calculations. But other than observed for the complexes adsorbed to the gold surface by Cattaneo and co-workers, the energetically most favorable alignment and the experimentally found dominating adsorption mode has the chloride ligand facing the substrate. This suggests that in contrast to the gold surface in the silver case, the interaction between the molecule and the surface is governed by both, first, the sulfur anchors and second also by the chloride ligand. The latter might be less dominant for the gold surface, thus explaining the different most favorable adsorption configurations. Furthermore, the differences may be related to distinct energetic preferences for specific M-S-C angles when thiolates are bound to gold (M = Au) or silver (M = Ag) surfaces^31^.

However, for potential application in electrochemical CO_2_ reduction, which requires chloride dissociation for substrate (CO_2_) binding, this adsorption configuration appears to be unfavorable as it sterically hinders the binding of incoming substrate. Hence, realization of the standing-up configuration seems to be worth investigating. It should be noted, though, that relative energies of different configurations for the immobilized molecules may be significantly altered by the local electric fields at the electrode-solution interface during electrocatalysis. While it remains unclear what are the implications for catalysis of the preferential nucleation of single molecules (Rebpy^S-S^) vs. clusters of molecules (Rebpy), one may assume the latter to allow for cooperative bimetallic reactivity on the surface, which may be either beneficial in case of synergistic bimetallic substrate activation^32^ but also detrimental by enabling bimolecular decomposition pathways^33–35^.

Future atomic scale studies for the investigation of the surface anchoring may involve the following strategies, which are currently pursued in our laboratories. Firstly, for the gold surface it has been qualitatively shown that the standing configuration can be realized^22^. We suggest a quantitative investigation of the efficiency of the sulfur anchors to generate the adsorption configuration promising for potential application in CO_2_ reduction catalysis. Secondly, we consider the sulfurated derivative of the related molecular catalyst Mo(bpy)(CO)_4_ an interesting molecule candidate, which does not inherit the chloride ligand^36^ and thereby allows to further investigate the contribution of the CO-surface interaction to the molecular anchoring.

## Methods

### Sample preparation

The synthesis of the complex Rebpy^S-S^, depicted in the inset of Fig. 1b, has been described elsewhere^21^. We deposited sub-monolayer films of the complex through thermal sublimation from a home-built molecular source onto Ag(001) that was cleaned by repeated Ar^+^-ion sputtering and annealing cycles. The source for the surface science experiments is sketched as inset of Fig. 2a and shown on the photos in Supplementary Fig. 1. For sample preparation a current is fed through the tungsten wire in order to heat the miniature test tube containing the molecules. In Fig. 2a, the currents used as well as the corresponding pressures inside the preparation chamber are shown. As evident by images of the molecule source taken at different times during sublimation, Supplementary Fig. 1, the color of the molecular material changes from bright orange at room temperature to dark red at more than 420 K as a consequence of the heating. After cooling down of the source the color returns to its original appearance.

### Scanning tunneling microscopy

Surfaces were studied in a home-built STM operating at temperatures of 77 K and under UHV conditions with pressures *p* < 5 × 10^−11^ mbar. Imaging was done in constant current mode, for better readability in the main text the constant current topographies are referred to as topographies. Minimal data processing was applied to the STM data such as plane fits to physically flat areas, subtraction of line averages, data interpolation, and application of, e.g., 3 × 3 median filters. All interpreted features are present in the raw data and do not originate from image processing.

### Density functional theory

We performed DFT calculations using the Vienna Ab initio Simulation Package^37–39^, version 6.3.0. All DFT calculations utilized a plane wave basis set, the projector-augmented wave method^40,41^, and the nonlocal optB86b-vdW functional^42^ to account for dispersion interactions. The optB86b-vdW functional was chosen since it is particularly suitable for absorption calculations of molecules on surfaces^43–46^. A plane wave energy cutoff of 400 eV was employed with a single Γ centered k-point due to the large size of the systems studied (convergence is shown in Supplementary Fig. 10). The electronic self-consistent loop was converged to 10^−6^ eV using no symmetry constraints (ISYM = 0) and high precision (PREC = Accurate). Gaussian smearing was used (ISMEAR = 0) for partial occupancies with the smearing width set to 0.05 eV. Since neither the Ag surface nor the Rebpy^S-S^ molecule is magnetic, non-spin-polarized calculations (ISPIN = 1) were performed.

The initial relaxation of the 3-layerd 7 × 7 × 1 Ag(001) surface was performed considering both the lattice and ionic degrees of freedom until the norms of all the forces were smaller than 0.01 eV/Å. For all relaxations of the Ag(001) surface with adsorbed molecules, only the ionic degrees of freedom were considered and the lattice parameters were kept fixed. The atoms in the bottom layer of the Ag(001) surface were kept fixed throughout all relaxations. The binding energy was calculated as ${E}_{b}={E}_{{tot}}-({E}_{{surf}}+{E}_{{mol}})$, where ${E}_{{surf}}$ is the energy of the clean Ag(001) surface, ${E}_{{mol}}$ is the energy of the molecule in vacuum and ${E}_{{tot}}$ is the energy of the combined system.

To generate simulated STM images for the fully relaxed structures, we employed the Tersoff-Hamann scheme^30^ with partial charge densities (LPARD = .TRUE) evaluated for all bands within 1 eV of the Fermi level (NBMOD = −3 and EINT = 1.0 0.0). Constant current STM images were generated using the partial charge densities obtained from the DFT calculations and a custom Python script. The Python script calculates the z-height where the tunneling current is constant using the expression $I=\,\frac{1}{40}{n}^{2}$ from ref. ^47^, where $n$ is the charge density and $I$ is the tunneling current set at 50 pA.

### Supplementary information

Supplementary Information

## Data Availability

The data that support the findings of this study are available from the corresponding author upon reasonable request.
