# Supplementary material for: Testing functional anchor groups for the efficient immobilization of molecular catalysts on silver surfaces
Source: Commun Chem. 2024 May 10;7:107. doi: 10.1038/s42004-024-01186-3 (PMC11082172; doi:10.1038/s42004-024-01186-3)
Supplement: Supplementary file 1 — Supplementary Information [file 42004_2024_1186_MOESM1_ESM.pdf]

Supplementary Information

Testing functional anchor groups for the efficient immobilization of molecular catalysts on silver surfaces

Ole Bunjes[1], Alexandra Rittmeier[1], Daniel Hedman[2], Shao-An Hua[3], Lucas A. Paul[3], Franc Meyer[3], Feng Ding[2,4], and Martin Wenderoth[1*]

Email: * martin.wenderoth@uni-goettingen.de

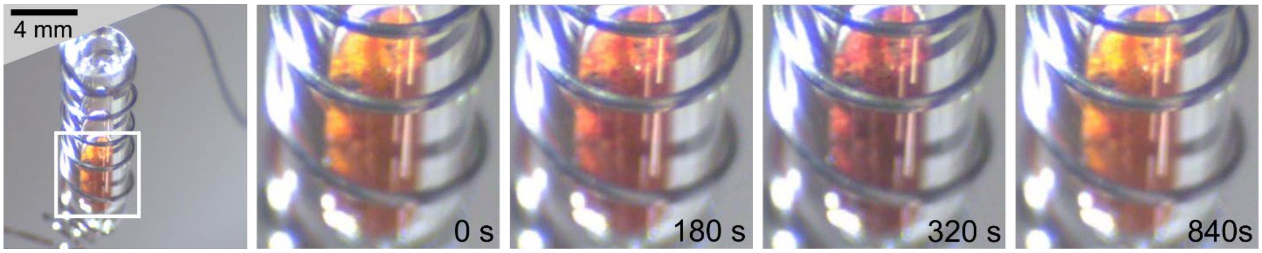

Supplementary Figure 1: Photo of molecule source with four close-ups of the region indicated by the white square that were taken at different times during the course of the sublimation process.

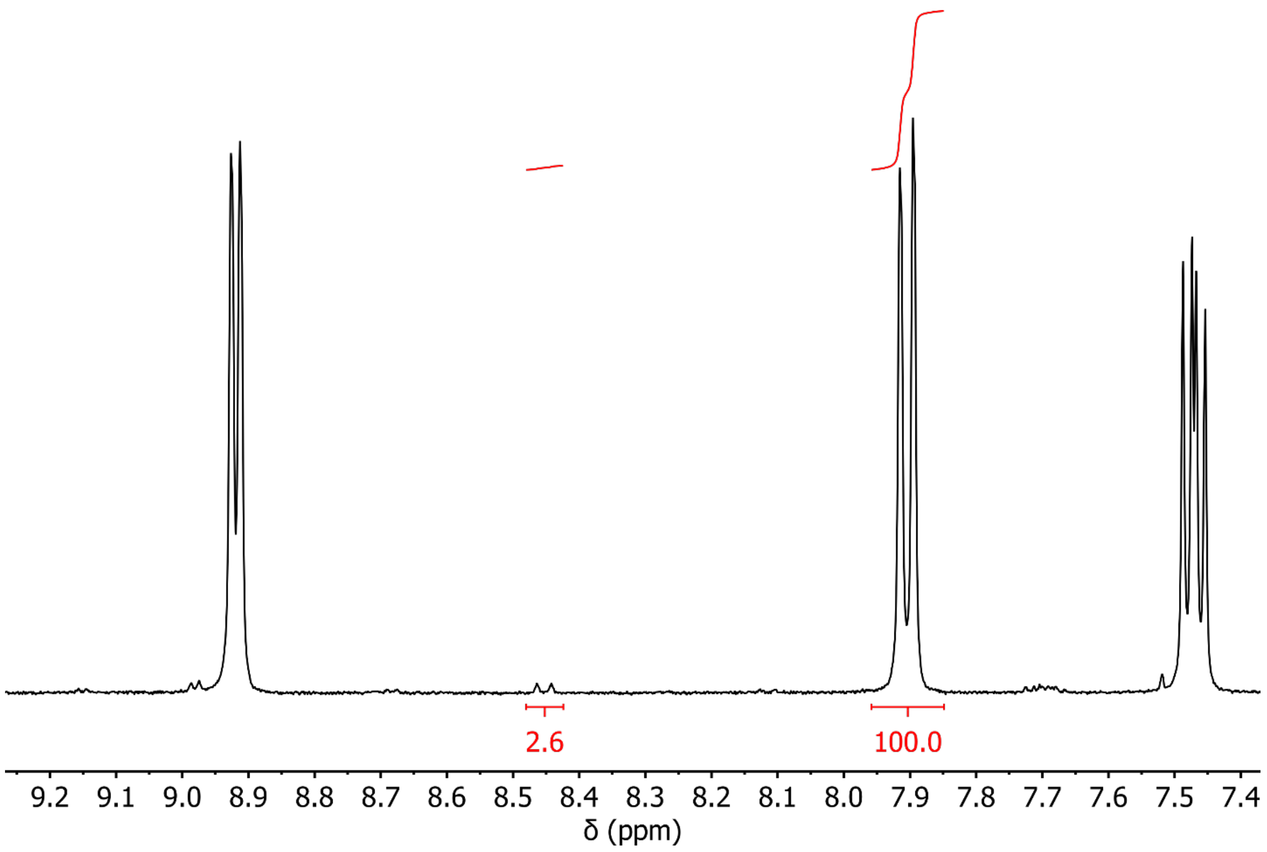

Supplementary Figure 2: High-resolution version of ^{1}H NMR spectrum (solvent $CDCl_3$) shown in dark red in Fig. 2(c). Integration of the spectrum for quantitative evaluation is shown in red.

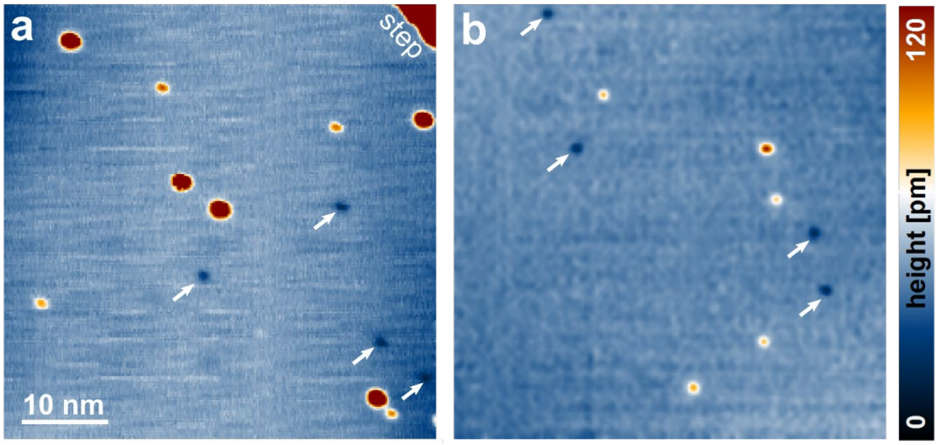

Supplementary Figure 3: Origin of defects on crystal surface. (a) Crystal side covered with molecules (dark orange, please note these molecules do show a different contour as the asymmetric appearance discussed in this manuscript), $U_{bias} = 2$ V, $I_{set} = 50$ pA. (b) Crystal side that was shadowed during deposition, hence without molecules, $U_{bias} = 1$ V, $I_{set} = 50$ pA. White arrows indicate depression-like defects, which could be sulfur atoms. Both sides show comparable defect densities (bright and dark signatures). Therefore, it is unlikely that the defects originate from the deposition of the molecules, supporting the molecules to stay intact upon arrival on the surface. Instead, the defects may originate from a different cause, e.g. the sample preparation procedure.

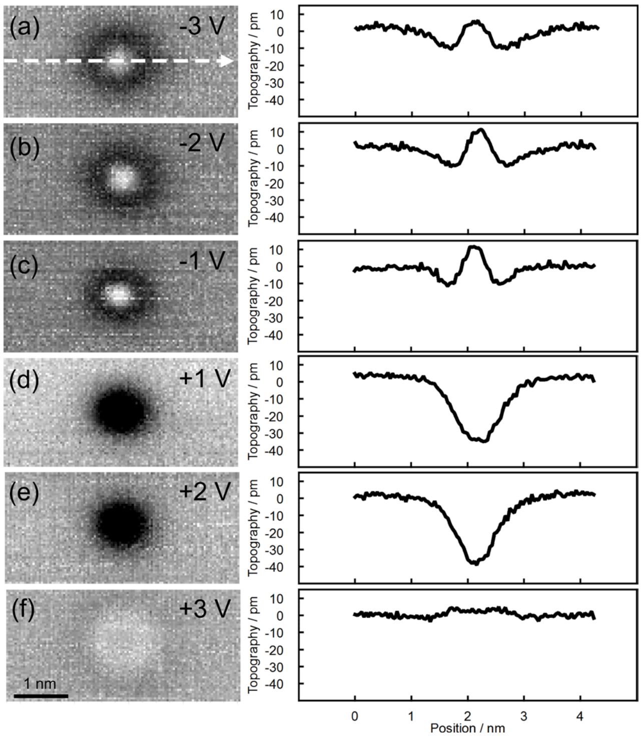

Supplementary Figure 4: Investigation of a single point defect. (a-f) Topographic signature of surface defect under varying bias voltage. Line profiles are taken through the center of the defect as exemplarily indicated by the dashed white arrow in (a), I_{set} = 50 pA. This change of contrast observed with inverting bias voltage fits to the behavior observed for sulfur impurities on the Ag(001) surface in the literature [1].

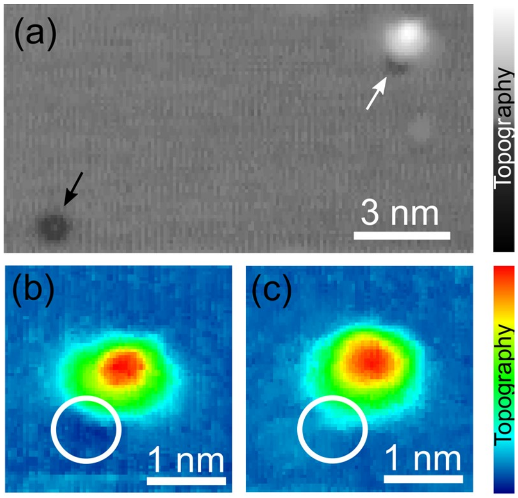

Supplementary Figure 5: Defect next to asymmetric molecular contour appears similar to the ones, which are likely sulfur impurities. (a) “Isolated” asymmetric molecular contours (top right hand corner) are always close to a surface defect (white arrow). The topographic appearance of these defects is similar to surface defects (black arrow), which are likely sulfur atoms (Supplementary Fig. 4). U_{bias} = +1 V, I_{set} = 50 pA. (b) At positive bias voltage the defect next to the molecule appears as depression (encircled in white), U_{bias} = +1 V, I_{set} = 50 pA. (c) At negative bias voltage the depression (white circle) vanishes, U_{bias} = -1 V, I_{set} = 50 pA, this qualitatively agrees with the observations made for sulfur atoms. However, it is fair to say that we cannot be fully sure about the nature of these defects, since firstly, the close proximity of the molecule could alter the defect’s topographic appearance and secondly, STM is not a chemically sensitive method.

Supplementary Note 1:

Statistics of defects on Ag(100):

In order to directly compare the density of intrinsic defects with possible molecule-induced defects, we prepared a sample that is only half covered with molecules. On this sample we had a mean density of 14.8 defects (+-3.6) on 100x100 nm^2 on the molecule-covered side. On the uncovered side we found a defect density of 15.6 (+-4.5) on 100x100 nm^2.

Statistics of molecules on the free surface:

We have analyzed samples with an estimated coverage on the terrace of 2.4 (± 0.3) 10^{-3} and 6.7 (± 0.5) 10^{-3} molecules/nm^2. In total we have counted more than 2000 molecules. About 90 % are found to be bound to a step.

We analyzed 31 clusters based on two molecules (which is 72% (±13%) of all clusters). We observed 7 clusters based on three molecules (16% (±9%) of all clusters) and 3 clusters based on four complexes (7% (±4%) of all clusters). Clusters of more molecules were observed only two times.

Regarding isolated molecules on the surface, we found 177 single free molecules, from which 8 molecules show an asymmetric constant current topography. In this survey sample, all 8 molecules had a defect nearby.

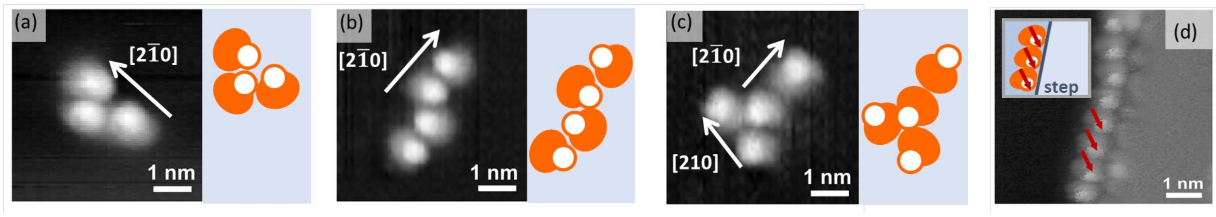

Supplementary Figure 6: Comparison of experimental data and the proposed orientation of the molecules. The images show exemplary constant current topographies and the extracted anisotropy, indicated by the orange sketches.

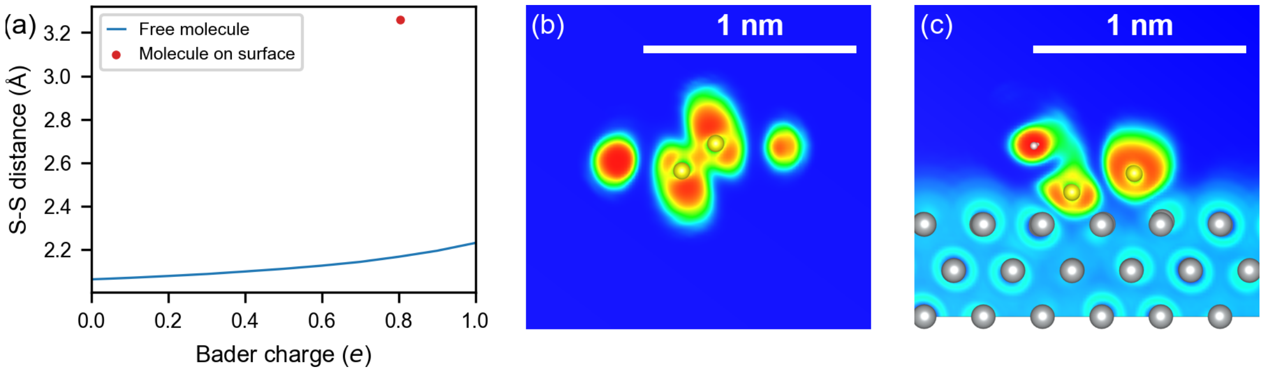

Supplementary Figure 7: Bonding and charge transfer for Rebpy$^{S\text{-}S}$. (a) S-S distance as a function of Bader charge for the Rebpy$^{S\text{-}S}$ as a free molecule (blue line) and on the Ag(001) surface (red dot). Please note, that the Bader charge of the molecule is scaled in units of elementary charge e. The Bader charge is positive which means that the molecule has become charged, i.e., gained electrons which makes it negatively charged.

Electron localization function (ELF) of the free molecule (b) and of the molecule adsorbed on the Ag(001) surface (c). The free molecule in the simulation box is charged by adding electrons to the DFT calculation. As the molecule becomes more charged the S-S distance increases as the S atoms becomes negatively charged. Yellow and grey circles indicate the positions of sulfur and silver atoms, respectively. The ELF is color coded, blue represents highly delocalized electrons (ELF=0) and red represents highly localized electrons (ELF=1). The change in ELF between the two sulfur atoms (red/orange for free molecule in Supplementary Fig. 6(b) and blue for adsorbed molecule in Supplementary Fig. 6(c)) indicates breakage of the S-S bond for the adsorbed molecule. Upon adsorption charge transfer occurs between the silver substrate and the molecule, cf. (a), and this charge transfer weekends the S-S bond (the S-S distance increases). However, the charge transfer alone is not enough to cause the S-S bond breakage (large S-S distance) seen when the molecule is adsorbed on the substrate. We attribute this to the strong covalent interaction between the S and Ag atoms as seen in the ELF in Supplementary Figure 6(c). This in addition to the charge transfer causes energetically favorable S-S bond breakage once the molecule is absorbed on the substrate.

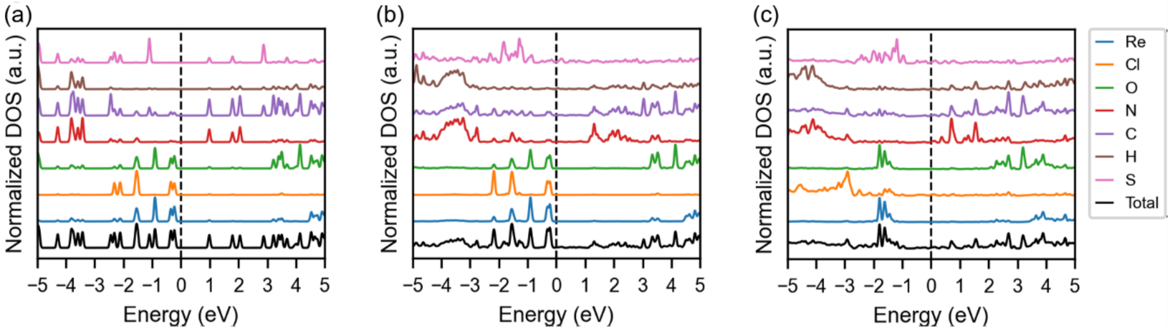

Supplementary Figure 8: DFT calculated density of state for the Rebpy$^{S\text{-}S}$ in case of free molecule (a) and adsorbed on the Ag(001) surface with sulfur anchors facing the substrate (b) and the Cl ligand facing the substrate (c). Here 0 represents the Fermi level.

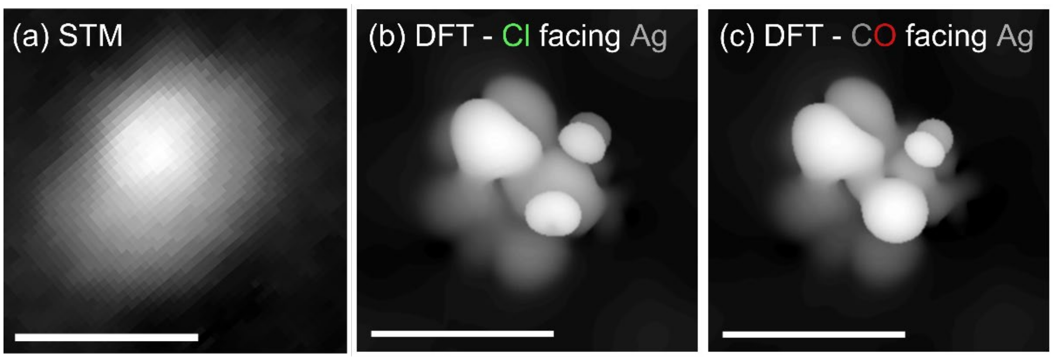

Supplementary Figure 9: Side-by-side comparison of experimental data and DFT simulated constant current topographies. (a) Rotated topography from Fig. 3(a). (b+c) Simulated topographies from Fig. 4(c+f). In all three images white corresponds to the topographic maxima. The scale bars have the length of one nanometer.

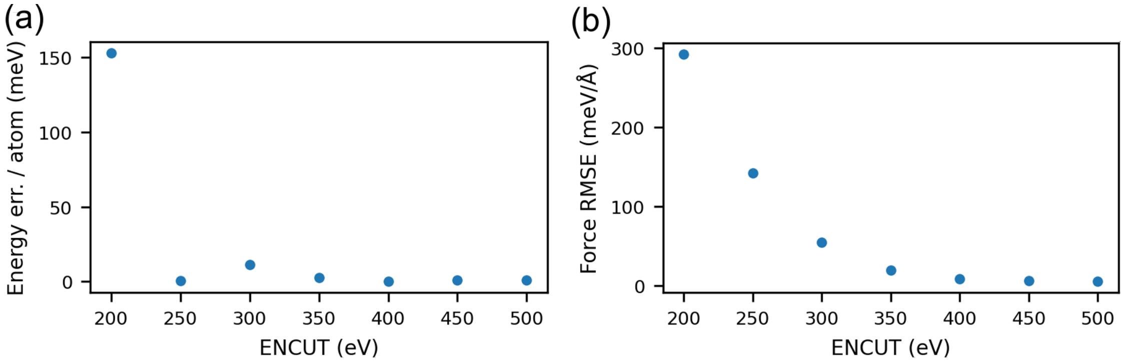

Supplementary Figure 10: DFT convergence study with respect to plane wave energy cutoff. (a) DFT energy error and (b) DFT force RMSE as a function of plane wave energy cutoff. Here the error was calculated by comparing the energy and force with a reference calculation performed at high plane wave energy cutoff (800 eV).

Supplementary Reference

[1] P. M. Spurgeon et al., “Characteristics of sulfur atoms adsorbed on Ag(100), Ag(110), and Ag(111) as probed with scanning tunneling microscopy: experiment and theory,” Phys. Chem. Chem. Phys., vol. 21, no. 20, pp. 10540–10551, 2019.
